# Formalin Fixation at Low Temperature Better Preserves Nucleic Acid Integrity

**DOI:** 10.1371/journal.pone.0021043

**Published:** 2011-06-15

**Authors:** Gianni Bussolati, Laura Annaratone, Enzo Medico, Giuseppe D'Armento, Anna Sapino

**Affiliations:** 1 Department of Biomedical Sciences and Human Oncology, University of Turin, Turin, Italy; 2 Department of Oncological Sciences, Institute for Cancer Research and Treatment (IRCC), University of Turin, Candiolo, Italy; University of Hong Kong, Hong Kong

## Abstract

Fixation with formalin, a widely adopted procedure to preserve tissue samples, leads to extensive degradation of nucleic acids and thereby compromises procedures like microarray-based gene expression profiling. We hypothesized that RNA fragmentation is caused by activation of RNAses during the interval between formalin penetration and tissue fixation. To prevent RNAse activation, a series of tissue samples were kept under-vacuum at 4°C until fixation and then fixed at 4°C, for 24 hours, in formalin followed by 4 hours in ethanol 95%. This cold-fixation (CF) procedure preserved DNA and RNA, so that RNA segments up to 660 bp were efficiently amplified. Histological and immunohistochemical features were fully comparable with those of standard fixation. Microarray-based gene expression profiles were comparable with those obtained on matched frozen samples for probes hybridizing within 700 bases from the reverse transcription start site. In conclusion, CF preserves tissues and nucleic acids, enabling reliable gene expression profiling of fixed tissues.

## Introduction

Since its introduction as a histological fixative back in the 19^th^ century [1], the 4% formaldehyde solution in water (formalin) has been adopted as the fixative of choice in histopathology. However, the uses of formalin-fixed tissues have varied over time. Originally, optimal morphological preservation was the sole requirement, but in more recent times, with the advent of immuno-histochemical typing, reliable antigenic preservation is also required [2], [3]. As a consequence, the protocols of formalin fixation have become stricter. This issue is particularly relevant in onco-pathology for the evaluation of factors predicting responsiveness to therapeutic treatments, and thus, fixation in phosphate buffered formalin (PBF) of breast cancer tissue blocks for no less than 6 and no more than 48 hours is now required in order to guarantee an optimal evaluation of Estrogen (ER) and Progesterone Receptors (PgR) and HER2 expression [4], [5].

In more recent times, a crucial request in cancer pathology has been nucleic acid preservation from formalin-fixed paraffin-embedded (FFPE) tissues because large tissue archives would thus be available for gene expression profiling, with the goal of generating new and reliable diagnostic and prognostic parameters [6], [7]. The first and most crucial issue that must be addressed for molecular gene signatures is ensuring that the sample is properly collected to obtain good quality RNA. The accuracy of molecular tests is utterly dependent on careful preservation of biological samples prior to analysis and then on an adequate sampling of fresh tissues that have to be representative of and enriched for the tumor cell population in order to obtain the specific RNA target. Studies conducted on the preservation status of nucleic acids in FFPE tissues generally agree on the relatively good (though not optimal) preservation of DNA [8]. On the contrary, RNA has been found to be heavily degraded and fragmented so that only short sequences (approximately 100–200 nucleotides) can be recognized and amplified [9]–[13]. The reasons for this effect are presently unknown, but some hints can be derived from the numerous studies conducted on the reaction of formaldehyde with different tissue components and specifically with nucleic acids [14]–[16].The main effect of formaldehyde in tissues is linked to the formation of methylol groups on amino groups first, followed by the establishment of cross-linking methylene groups that lead to proper fixation [13]. Bases of nucleic acids are involved in this process, resulting in cross-linking with side-chain amino groups of proteins. However, this linkage is at least partly reversible following extensive treatment of FFPE tissue sections with peptidases and high temperature [9], [13], [17], [18]. We conclude that cross-linking of nucleic acid bases cannot be the sole responsible for nucleic acid fragmentation and degradation. Chung YJ et al. [9] have demonstrated that substantial RNA degradation may occur during the so called “warm ischemia” that refers to the time of transfer from an operation room (or removal of blood supply) to pathology laboratory. The RNA degradation due to warm ischemia may be slowed down by cooling the specimen. The ischemic process may continue during fixation [9]. In fact, while formalin penetration is a rather fast process, tissue fixation is known to be a slow process that requires long time exposure [16], [18]. Another suggested mechanism of RNA degradation in FFPE tissues is incomplete dehydration from inadequate tissue processing [9].

Based on these hypotheses, we have further considered that RNA degradation would be inhibited by maintaining low temperature through all the process of fixation. The aim of the present study was thus to evaluate whether a protocol based on processing tissues with formalin at low temperature would better preserve nucleic acid integrity, while preserving morphological and antigenic features as well. The presently described cold fixation (CF) process in formalin was experienced on several human specimens, specifically in colon and breast cancers.

## Results

### Morphology and antigen preservation

No distinction was observed in morphology between tissues processed either using standard formalin fixation at room temperature or CF (4°C) method, with blinded pathologists opting in 7 out of 10 cases for the latter as better preserved. No shrinking or distortion artifacts were observed (see Figure S1). No appreciable difference was observed in immunohistochemical reactivity for the markers (ER, PgR, Ki67, HER2, CEA, CK20) tested.

### RNA preservation

The RNA quality as initially assessed by lab-on-chip analysis showed that in the control fresh-frozen specimens, the RIN (RNA Integrity Number) value ranged between 8.5 and 7.2. Values were much lower (RIN ranging between 3.80 and 2.20) in RNA samples obtained from paraffin sections of CF-treated tissues. As expected, after standard formalin fixation the RIN values were not measurable.

**Figure 1:**
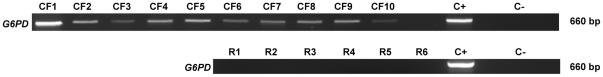
RT-PCR for G6PD on cold-fixed (CF 1–10) and routine FFPE (R 1–6) samples. A specific signal for G6PD was detected at 660 bp in CF-treated tissues.

The RT-PCR study for homeobox genes expression was conducted in parallel on fresh frozen, CF-treated and standard FFPE samples of 10 cases with amplification of the *HMBS/ABL1/B2M* genes (128 bp, 193 bp and 334 bp in length, respectively) and of *G6PD* (660 bp). The CF processing allowed the amplification of all genes up to 660 bp, whereas RNA extracted from standard-fixed specimens allowed the amplification of the shorter segments (128 bp and 193 bp) only (Figure 1).

We used the amplification of segments of *CK20* RNA of different length (329 bp, 500 bp and 716 bp) as a tissue-specific marker of colon cancer specimens. The amplification of all the *CK20* RNA segments (up to 716 bp) was obviously successful with RNA extracted from all of the frozen samples, while RNA segments were successfully amplified only in 3 of the standard-fixed tissues and the fragments were no more than 329 bp in length. In all the CF-treated specimens, RNA amplification was consistently successful at 329 and 500 bp, and also occasionally at higher lengths (716 bp).

The expression of the mammaglobin gene (331 bp) was tested in the four samples of breast cancer tissue and the *ER* gene (349 bp) in the three cases showing ER protein expression by immunohistochemistry. The RT-PCR tests were consistently positive in frozen and CF-treated samples, whereas in standard-fixed tissues, mammaglobin, but not *ER* was positive in 2 out of four cases.

### Microarray analysis

**Figure 2:**
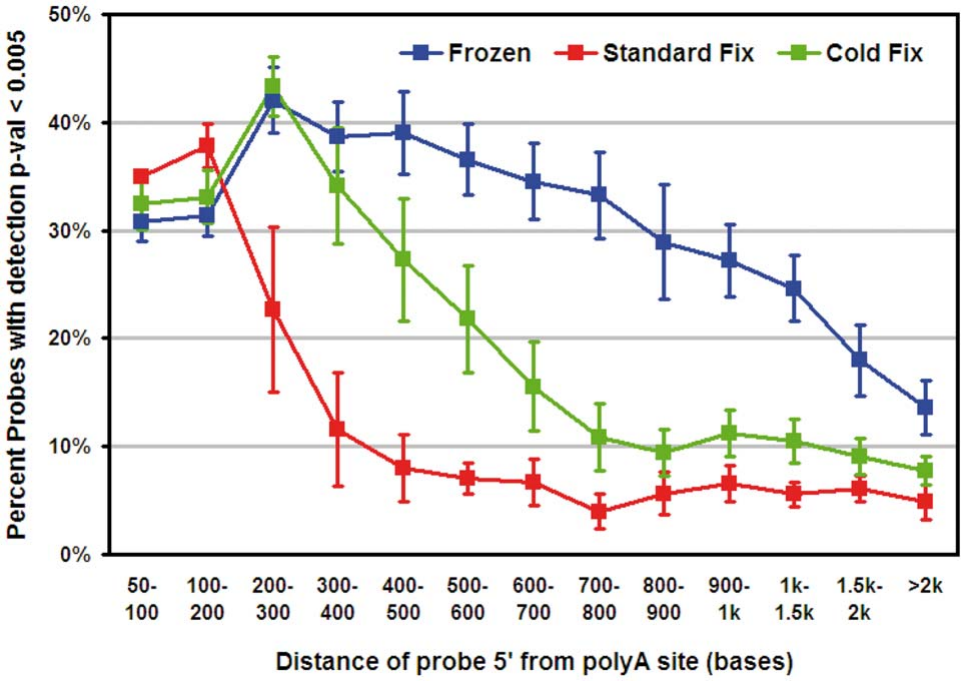
Messenger RNAs from cold-fixed samples are detected by microarray probes hybridizing more than 500b. Messenger RNAs from cold-fixed samples are detected by microarray probes hybridizing more than 500b upstream from the reverse transcription start site. Graph showing the fraction of probes with detectable signal (y-axis) for each bin of distance of target sequence from the mRNA poly(A) site from which RT is initiated (x-axis).

The standard protocol for Illumina Beadarray gene expression analysis was employed on RNAs obtained from frozen, CF and standard-fixed samples. This protocol employs a T7-oligo-dT primer for cDNA synthesis, so that reverse transcription (RT) starts from the beginning of the poly(A) tails. Therefore, if the RNA is fragmented, an array probe gives reliable signal only if the distance of its target sequence from the poly(A) site is shorter than the average length of the RNA fragments. To annotate the probes of the Human HT-12v4 BeadArray with their distance from the poly(A) site, we blasted all of them against the human RefSeq database, containing well-annotated mRNA transcripts. In total, 18470 probes were found to univocally match one RefSeq transcript, and were subdivided into 13 bins based on the distance of their target sequence from the transcript poly(A) site (from 50–100 bases to more than 2000 bases). For each bin, the fraction of probes giving reliable expression signal (detection p-value<0.005) was calculated. This analysis is summarized in Figure 2. While RNAs from standard-fixed samples yielded, as expected, a sufficient fraction of detection only for probes binding up to 200b from the poly(A), RNAs from cold-fixed samples gave a significantly better profile, with a good fraction of detection for probes binding also 500–700b from the poly(A). Figure S2 shows tissue-specific analysis of cancer samples from colon (four), breast (four), pancreas (one) and stomach (one). For all colorectal cancer samples, a replicate cold fixation was performed and RNA was extracted. Overall, fragment length of RNA from cold-fixed samples was comparable for all tissues, and reproducible within tissue and within sample, with slightly shorter fragments only for pancreatic cancer.

**Figure 3:**
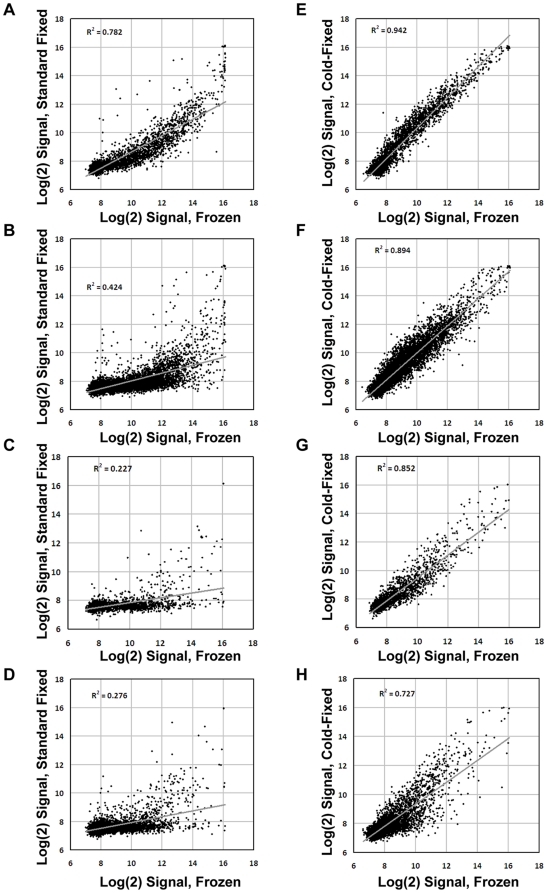
Frozen and cold-fixed samples generate highly correlated expression profiles. Dot plots comparing signal intensities of frozen vs standard-fixed tissues (A–D) or frozen vs cold-fixed tissues (E–H). The plots show four groups of probes based on the distance of their target sequence from the RT start site: less than 200 bases (A,E) 200–500 bases (B, F) 500–700 bases (C, G), more than 700 bases (D, H).

**Figure 4:**
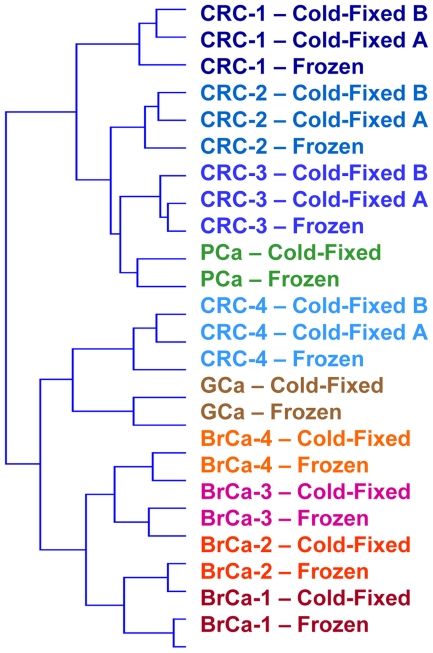
Frozen and cold-fixed samples from the same specimen cluster together. Hierarchical clustering of samples based on 12526 microarray probes hybridizing up to 700 bases from the poly(A) site. CRC, Colorectal cancer; PCa, Pancreatic cancer; GCa, Gastric cancer; BrCa, Breast cancer.

We then tested the correlation between gene expression profiles obtained from frozen and fixed samples of the same tissue specimen. Using all probes, the average Pearson correlation between frozen and standard fixed or cold-fixed samples was respectively 0.615±0.087 and 0.894±0.044. Interestingly, the average Pearson correlation between two cold-fixed samples from the same tissue was extremely high (0.984±0.02). Individual pairwise correlations and representative dot plots are provided in Figure S3. The same correlation analysis was then run using probes binned for their distance from the poly(A) site. Standard fixed samples displayed acceptable correlations with their frozen counterparts only for probes binding up to 200 bases from the RT start site (Figure 3 A), while for further upstream probes the correlation dropped to unacceptable levels (Figure 3 B–D). Conversely, CF samples displayed highly correlated gene expression profiles for probes binding up to 700 bases from the poly(A) (Figure 3 E–G), with reasonable correlation still present for probes more than 700 bases-away from the RT start site (Figure 3 H). Detailed binned correlation analysis for all sample pairs is provided in Figure S4. Finally, we verified if technical artifacts deriving from the CF procedure could impair comparability between gene expression profiles of CF and frozen samples from the same specimen. To this aim, we performed data clustering using 12526 probes mapped within 700 bases from the poly(A) site. For each probe, the signal was averaged across all samples, and the Log(2) ratio was calculated against this average for each frozen and CF sample. Hierarchical clustering with cosine correlation was performed with the GEDAS software [19] and showed a tight co-clustering of frozen and CF samples from the same specimen (Figure 4). Overall, gene expression profiling analysis showed that RNAs from CF samples are significantly less fragmented that those from standard FFPE samples, with high amounts of more than 500-base long fragments.

### DNA preservation

DNA study by FISH performed on the breast cancer specimens using *HER2* gene as a marker showed identical result in the CF-processed and matched standard-fixed samples.

Using QIAamp DNA minikit and PCR analyses, *CK20* DNA fragments at 755 bp length were assessable in the four colon cancer cases in parallel in fresh-frozen, standard-fixed and CF fixed specimens.

## Discussion

Following the demonstration that low temperature better preserves RNA, we have designed a protocol that considers keeping the specimen at 4°C from the surgical excision up to formalin fixation and the first step of alcohol dehydration. According to Fox and colleagues [15], there is a paradox in the working mechanism of formaldehyde because it penetrates tissues rapidly, but it fixes them very slowly. During this lapse of time, RNA degradation may occur as a consequence of warm ischemia [9]. In the present experiments, we extended the immersion time up to 24 hours at 4°C, thus limiting RNAse activity and allowing complete penetration by formalin and proper fixation of the tissue.

At the end of this “cold penetration” period, methylene glycol, the hydrated form acquired by formaldehyde in aqueous solutions, has already been establishing reactive groups, mainly with hydroxyl-methyl side groups of proteins, but the extensive formation of methylene cross-links, leading to proper tissue fixation, is a progressive and lengthy process [16]. Standard formalin fixation in about 24 hours, as currently practiced in routine histopathology, is therefore the result of a compromise between the request of a rapid diagnosis and a satisfactory morphological preservation. In the present study, CF-treated tissues were morphologically similar to standard-processed tissues: no stretching or cohartation artifacts were noticed (see Figure S1). The immunohistochemical reaction for the markers, which are routinely investigated in colorectal (CEA, CK20) and breast cancers (ER, PgR, Ki67 and HER2) gave results indistinguishable from those obtained from the samples of the same cases processed by standard method. However, the main objective of the present study was to preserve nucleic acid integrity. The preservation of DNA in FFPE tissues is known to be relatively good [6], [7] and it was not modified by CF. Major problems are instead related to successful extraction of usable RNA from FFPE samples. Farragher and colleagues [20] suggested that the limiting factors for RNA extraction from FFPE samples are: cross-linkage between nucleic acids and proteins, addition of monomethylol groups to the bases that modify the poly A tail inhibiting oligo primer annealing to the polyA tail and consequently the reverse transcription reaction and finally, the natural RNA degradation in the tissue prior to fixation due to RNAses activity. RNases are ubiquitous in the environment, and they are present in relatively high concentrations in many biological materials. Thus, the same authors suggest a change in clinical practice for the development of a standardised, consistent and short time interval between removal of specimen and fixation. In our experience, the poor preservation and fragmentation of nucleic acids currently observed in formalin-fixed tissues [6], [7], [10]–[13] can, at least partly, be prevented by keeping low temperatures before and during fixation. Our procedure includes in fact, the use of a vacuum to seal the tissue and facilitate the transfer at 4°C, which already prevents RNA degradation as previously demonstrated by our group [21].

Surprisingly, no studies are reported in the literature on the temperature of formalin as a variable and on the use of cold formalin fixation in order to preserve RNA in FFPE tissues. RIN analysis showed that significant RNA fragmentation still occurred in CF-processed tissues, but at a much lower extent than in standard-processed tissues with good results even in tissues obtained from pancreas and stomach that contain high level of hydrolytic enzymes. For example, the pancreas is rich in RNase (>1 mg RNase/1 g tissue) and is the source for most commercially produced RNase A (and its glycosylated derivative, RNase B). Recently, a gene expression assay (Whole-Genome DASL), capable of generating whole-genome gene expression profiles from degraded samples has been developed [22], [23], with amplicon sizes of 70–85 bp. At difference from these studies, the present work aims at improving RNA integrity of FFPE specimens, without changes of reagents or overall time of fixation. Indeed, RT-PCR amplification [10] of RNA fragments up to 660 bp from CF samples can be considered a notable improvement over room temperature formalin fixation. Gene expression analysis using a standard whole-genome microarray showed that mRNA profiles of CF-treated samples are comparable to those of fresh-frozen samples, which are, at present, the sole specimens considered suitable for microarray-based gene expression profiling. While RNAs from standard-fixed samples yielded, as expected, a sufficient fraction of detection only for probes binding up to 200b from the poly(A), RNAs from cold-fixed samples gave a significantly better profile, with good performances for probes binding also 500–700b upstream from the poly(A). It should be noted that in the case of the Illumina arrays used in this work, 12526 probes bind within 700 bases from the poly(A) site, which renders analysis of CF samples already genome-wide and reliable without need of dedicated probes or protocols.

Finally, the issue of adequacy of the material subjected to molecular analyses is of paramount importance. In routine practice, a significant proportion of molecular tests performed on fresh tissue samples are not successful due to incorrect sampling of the target area (for example no cancer cells in the sample). The present CF protocol overcomes these problems meeting the requirement of a correct identification of the material to be examined.

In conclusion, the presently proposed procedure of tissue conservation results in a definitely lower degree of mRNA fragmentation, while keeping the basic advantages that make formalin the fixative of choice in diagnostic histopathology. Although not yet reaching the nucleic acid quality obtained from fresh-frozen material, the CF approach represents a definite improvement in the degree of preservation of the molecular structures of FFPE tissues. Most importantly, this approach does not require profound changes in routine procedures currently adopted by most pathology laboratories, which renders its implementation feasible for standard practice. The possibility of obtaining high-quality mRNA from archival tissues opens prospects for wider gene expression profiling than presently feasible [24], [25].

## Materials and Methods

### Sample Collection Procedures

Following a procedure first published by our group [21] and now used as a standard in our hospital [26], four consecutive colon cancer, four breast carcinoma specimens, one case of pancreatic cancer and one case of gastric cancer were under-vacuum sealed in plastic bags using a semi-professional machine (Mod. VAC 10, by Milestone, Bergamo, Italy; see http://www.milestonemedsrl.com) inside the surgical theatre immediately after surgery, and kept at 4°C until transfer to the pathology laboratory. Once in the pathology lab the surgical specimens were processed without delay.

From each specimen, three samples were taken and processed as follows:

- *Standard fixation procedure (FFPE):* the sample (4 mm. thick) was fixed for 24 hours in 4% neutral-buffered formalin (NBF) (Histo-Line Laboratories, Milan, Italy) at room temperature (RT), routinely processed to paraffin embedding with an automatic processor (Leica ASP 300, Leica Microsystems, Wetzlar, Germany) and embedded in paraffin wax.
- *Cold fixation procedure (CF):* the sample was immersed in pre-cooled 4% NBF (Histo-Line Laboratories, Milan, Italy) at 4°C for 24 hours. Thereafter, the specimens were transferred in ethanol 95% at 4°C for 4 hours in order to keep nucleases inhibited during the time of ethanol penetration. The tissues were processed to paraffin embedding in the same processor (Leica ASP 300, Leica Microsystems, Wetzlar, Germany) and following the same program used for *FFPE*, but skipping the formalin and the first ethanol 95% steps.
- *Freezing procedure:* the sample was frozen in liquid nitrogen immediately after dissection and stored at −80°C.

**Figure 5:**
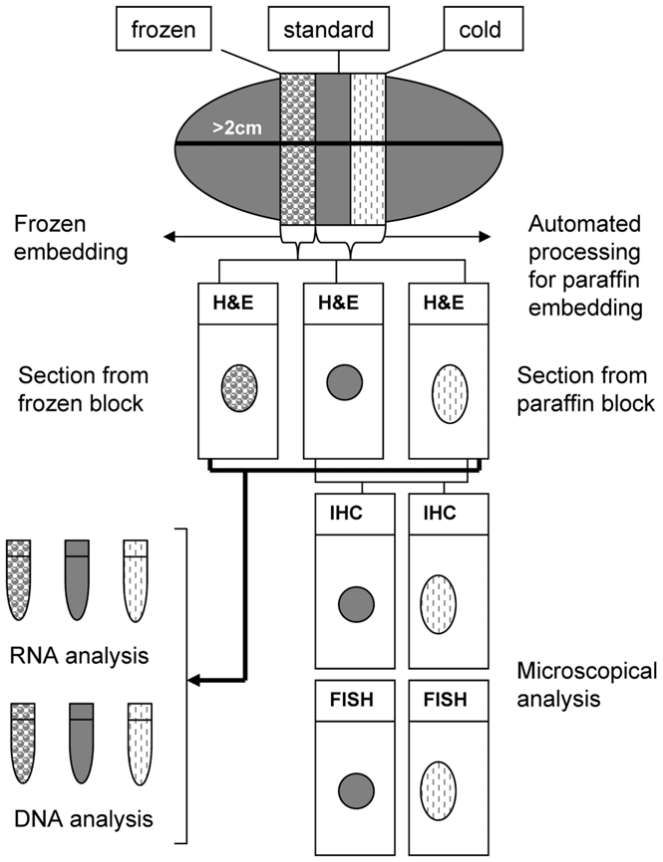
Study flowchart. Flowchart illustrating the design of the study.

The study was restricted to surgical specimens and to tumor larger than 2 cm. The patients were informed of the study protocol. Verbal consent for anonymous use of the tissue for study purposes was then obtained. In order to assure the diagnoses and tumor staging the frozen samples, cold and standard fixed samples were examined on hematoxylin and eosin (H&E) stained slides before proceeding to the experimental part. The design of the study (Figure 5) was approved by the ethic institutional review board for “Bio-banking and use of human tissue for experimental studies” of the Department of Biomedical Sciences and Human Oncology of the University of Torino. The same ethic institutional review board approved that written consent from the patients was not needed, given that the study did not interfere with diagnosis or treatment decisions. Data were analyzed anonymously.

### Staining, immunohistochemistry and Fluorescent *In Situ* hybridization (FISH) procedures

Sections 3 µm in thickness were cut from FFPE and CF blocks and routinely stained with hematoxylin and eosin (H&E). Immunohistochemistry was performed on tissue sections using an automated slide processing platform (Ventana BenchMark AutoStainer, Ventana Medical Systems, Tucson, AZ, USA) and the following primary antibodies: CK20 monoclonal mouse antibody (clone Ks 20.8, diluted 1∶100, DAKO, Glostrup, Denmark) and CEA monoclonal antibody (clone II-7 diluted 1∶100, DAKO, Glostrup, Denmark) to stain colon carcinomas; prediluted anti-ER rabbit monoclonal antibody (clone SP1, Ventana-Diapath, Tucson, AZ, USA), prediluted anti-PgR rabbit monoclonal antibody (clone 1E2, Ventana-Diapath), anti-Ki67 monoclonal antibody (clone MIB1, diluted 1∶100 Dako, Glostrup, Denmark) and anti-HER2 monoclonal antibody (clone 4B5, Ventana-Diapath, Tucson, AZ, USA) for breast cancer specimens. Positive and negative controls (omission of the primary antibody and IgG-matched serum) were included for each immunohistochemical run. FISH assay to evaluate *HER2* gene amplification was performed, following a previously reported protocol [27].

### RNA extraction

RNA was extracted from FFPE, CF and fresh frozen blocks. The number of sections needed to obtain a nucleic acid yield adequate for molecular analysis depended on the type of tissue samples (fibrosis, cellularity) and on the sample dimensions. On equal sample characteristics the number of sections was higher for paraffin embedded tissue because of the expected decrement in RNA and DNA yield from fresh to formalin fixed tissues. Sections were collected in a 1.5 ml sterile Eppendorf tube.

RNA isolation from the FFPE and CF samples was performed using the MasterPure Purification kit (Epicentre, Madison, WI, USA). Sections were deparaffinized by incubations in xylene followed by incubations in 100% ethanol. The ethanol was discarded, and the pellet was air-dried for several minutes at room temperature before Proteinase K treatment, according to the Chen optimized extraction “Method 3” for FFPE tissues [28]. RNA extraction from fresh frozen sections was performed with 1 ml of Trizol® reagent (Invitrogen, Carlsbad, CA, USA) according to manufacturer's instructions.

RNA pellets were resuspended in DEPC-treated water, and RNA concentrations were measured with a spectrophotometer (BioPhotomer Eppendorf AG, Hamburg, Germany). RNA samples were stored at −80°C until further analysis. The quality and quantity of the extracted RNA was also assessed using a Bioanalyzer 2100 (Agilent Technologies, Palo Alto, CA) based on a 28S/18S ribosomal RNA ratio and on the RIN.

### DNA extraction

DNA was isolated from FFPE, CF and fresh frozen blocks. The number of sections obtained for DNA analysis followed the same criteria reported for RNA extraction. Sections were collected in a 1.5 ml sterile Eppendorf tube and treated according to the manufacturer's protocol for the QIAamp DNA minikit (QIAGEN Ltd, Crawley, UK). The DNA concentration was measured spectrophotometrically, and the eluted DNA was stored at 4°C until further analysis.

### Reverse Transcriptase–Polymerase Chain Reaction (RT-PCR) and PCR Analysis

RT-PCR was performed after a DNAse treatment step with the TURBO DNA-free™ Kit (Ambion, Foster City, CA, USA). For each sample, up to 4 µg of RNA was reverse transcribed to cDNA with a High-Capacity cDNA Reverse Transcription Kit (Applied Biosystems, Foster City, CA, USA). RNA samples without Reverse Transcriptase were reverse transcribed as negative controls for DNA contamination in the PCR analyses.

Several tests were performed to evaluate the integrity of gene sequences of different length (see list and sequences in Table S1). Initially we investigated sequences of hydroxymethylbilane synthase (*HMBS*, NM_001024382.1, NM_000190.3), abelson kinase (*ABL1*, NM_005157.3, NM_007313.2) and beta2-microglobulin (*B2M*; NM_004048.2) genes, Cytokeratin 20 (*CK-20*; *KRT20*, NM_019010.1) (colon tissues), mammaglobin (*SCGB2A2*, NM_002411.2; 331 bp) and ER alpha (*ESR1*, NM_000125.3, NM_001122740.1, NM_001122741.1, NM_001122742.1; 346 bp) (breast samples) but finally, we performed a PCR for Homo sapiens glucose-6-phosphate dehydrogenase (*G6PD*, NM_000402.3, NM_001042351.1), resulting in a target sequence of 660 bp (primers sequences: FW 5′- ATCTTGGTGTACACGGCCTC-3′, REV 5′- CAACCACATCTCCTCCCTGT-3′).

CK-20 (*KRT20*, NT_010783.15) PCR amplification was also evaluated in genomic DNA samples (339 bp, 420 bp, 755 bp). The PCR conditions are reported in Table S1. Reactions were performed on PTC-100 Peltier Thermal Cycler (MJ Research, Inc., MA, USA), and the PCR products were separated by electrophoresis on ethidium bromide-stained 2% agarose gels. To reduce the risk of contamination from previously amplified products, separate areas were used for RNA isolation, amplification and electrophoresis.

### Microarray analysis

Biotinylated cRNA was prepared using the Illumina TotalPrep RNA Amplification Kit (Ambion, Inc., Austin, TX) according to the manufacturer's recommendations starting with 500 ng of total RNA. Hybridization of the cRNA to the HumanHT-12_V4 Expression BeadChip (Illumina, Inc., San Diego, CA), washing and scanning were performed according to the Illumina BeadStation 500× manual (revision C). Microarray data were summarized with the Beadstudio software (Illumina), and subsequently analyzed using Excel (Microsoft). The whole microarray dataset is MIAME compliant. Raw data are deposited in Gene Expression Omnibus (GEO Accession number GSE27175).

## Supporting Information

Figure S1 **Histology (H&E staining) of tissues processed either routinely (FFPE) or following the CF procedure.** For all samples: (**a**) Cold-Fixed (10×); (**b**) Cold-Fixed (20×); (**c**) Standard-Fixed (10×); (**d**) Standard-Fixed (20×).
(PDF)

Figure S2 **Probe detection analysis on cancer samples subdivided by tissue of origin.** (**a**) Four CRCs; (**b**) Four breast cancers; (**c**) One pancreatic and one stomach cancer. For all CRC samples, a replicate cold fixation was performed and RNA was extracted, to assess consistency of the procedure on the same tissue sample.
(PDF)

Figure S3 **Cold-fixed samples generate reproducible expression profiles, highly correlated with those generated by frozen samples.** (**a**) Pearson correlation between frozen (F) and Cold-Fixed (CF) or Standard-Fixed (SF) samples, plus correlation between replicate samples from the same cold-fixed tissue. (**b–e**) Dot plots comparing, expression profiles of RNA from, respectively: frozen vs standard-fixed tissue (**b**), Frozen vs Cold-Fixed tissue of a representative CRC sample (**c**), frozen vs Cold-Fixed tissue of a representative breast cancer sample (**d**), Cold-fixed vs replicate sample extracted from the same cold-fixed tissue (**e**).
(PDF)

Figure S4 **RNA from cold-fixed samples generates reliable expression profiles also with probes far away from the Reverse Transcription start site.** Pearson correlation was analyzed for subgroups of microarray probes, based on their distance from the transcript 3′-end. (**a**) Breast Cancer samples; (**b**) CRC samples; (**c**) Pancreas and stomach cancer; (**d**) CRC replicated cold-fixed samples.
(PDF)

Table S1 **RT-PCR conditions.**
(PDF)
