# Supplementary material for: Formalin Fixation at Low Temperature Better Preserves Nucleic Acid Integrity
Source: PLoS One. 2011 Jun 15;6(6):e21043. doi: 10.1371/journal.pone.0021043 (PMC3115967; doi:10.1371/journal.pone.0021043)

Figure S1: Histology (H&E staining) of tissues processed either routinely (FFPE) or following the CF procedure.

For all samples: **(a)** Cold-Fixed (10x); **(b)** Cold-Fixed (20x); **(c)** Standard-Fixed (10x); **(d)** Standard-Fixed (20x).

CRC-1

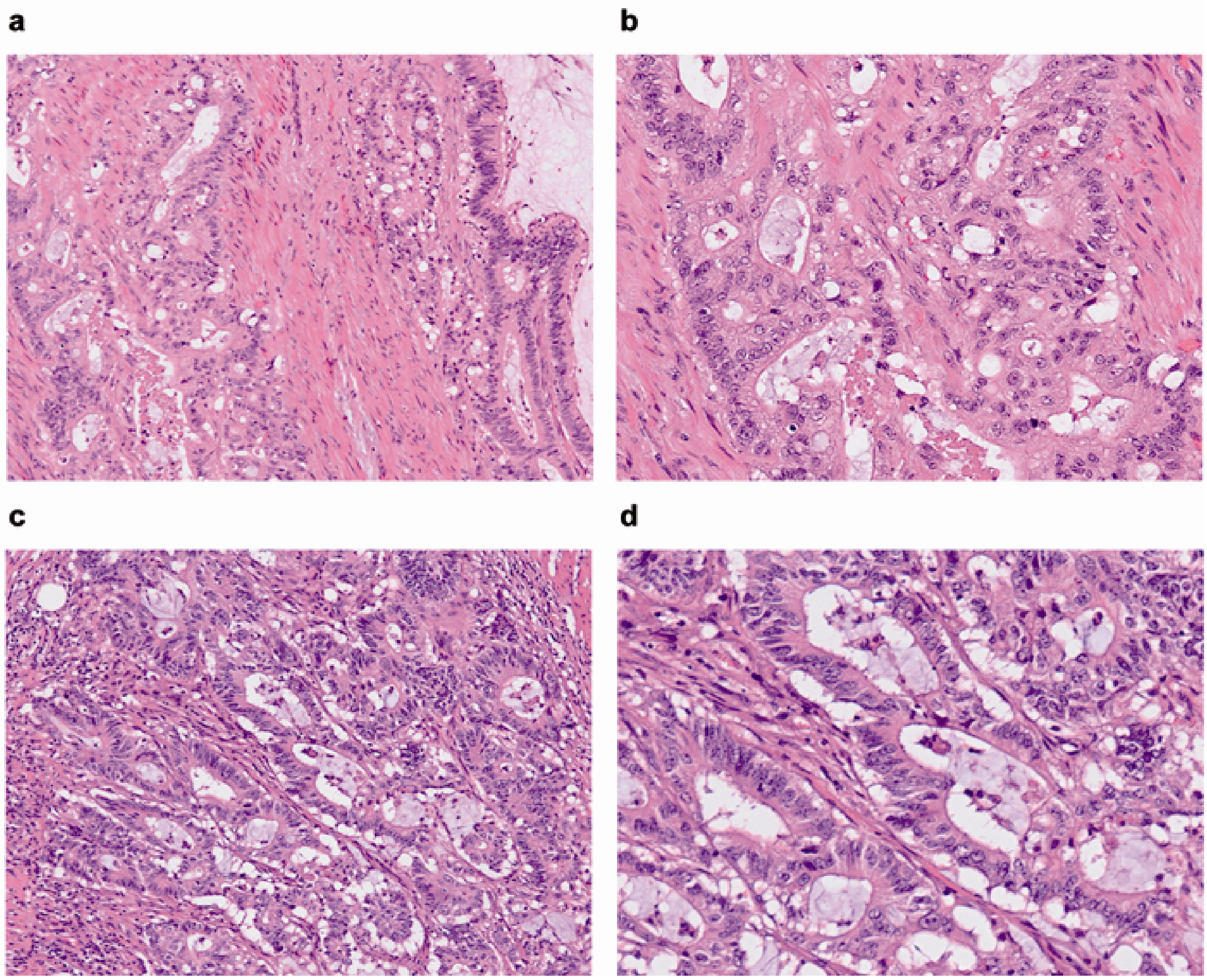

CRC-2

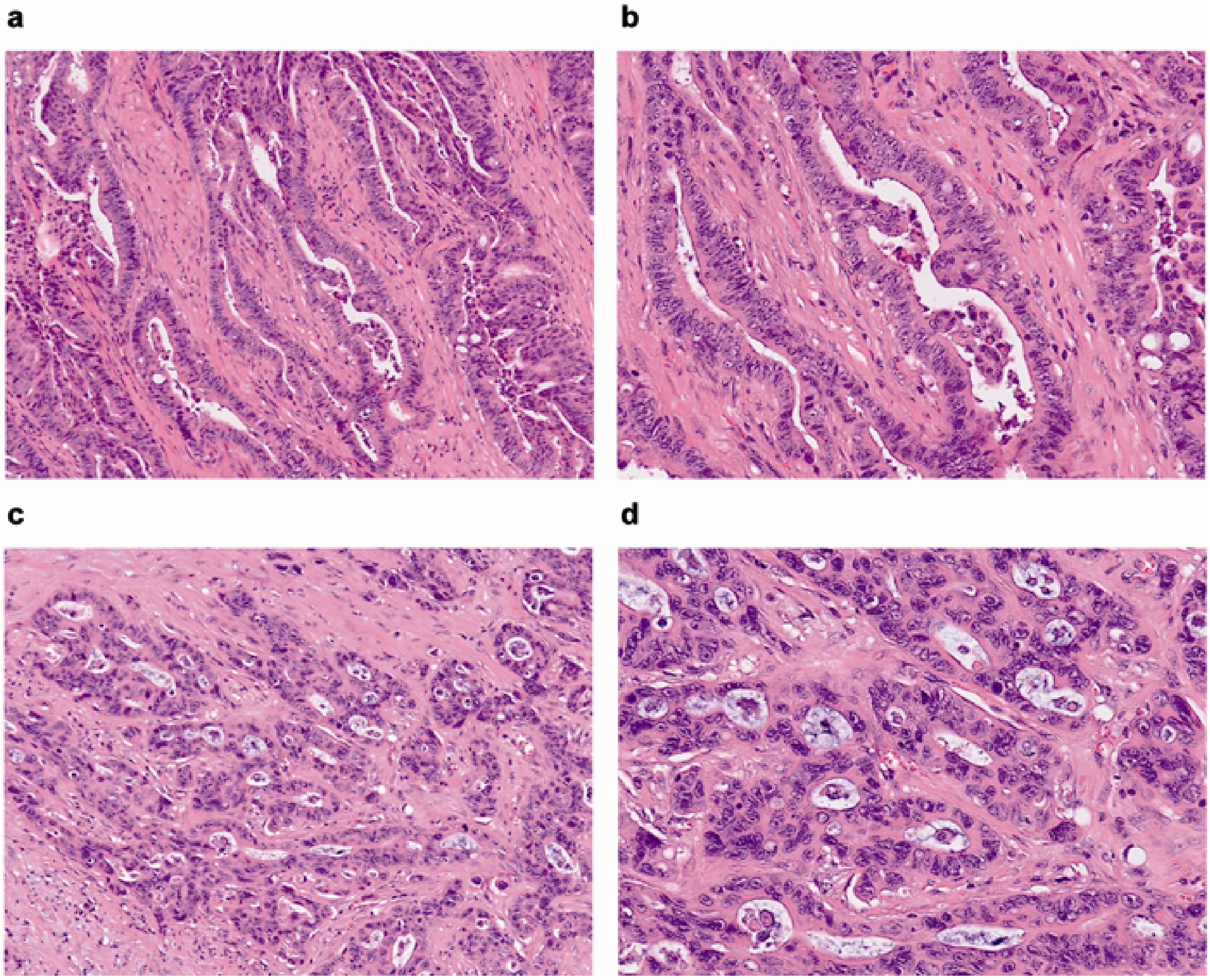

CRC-3

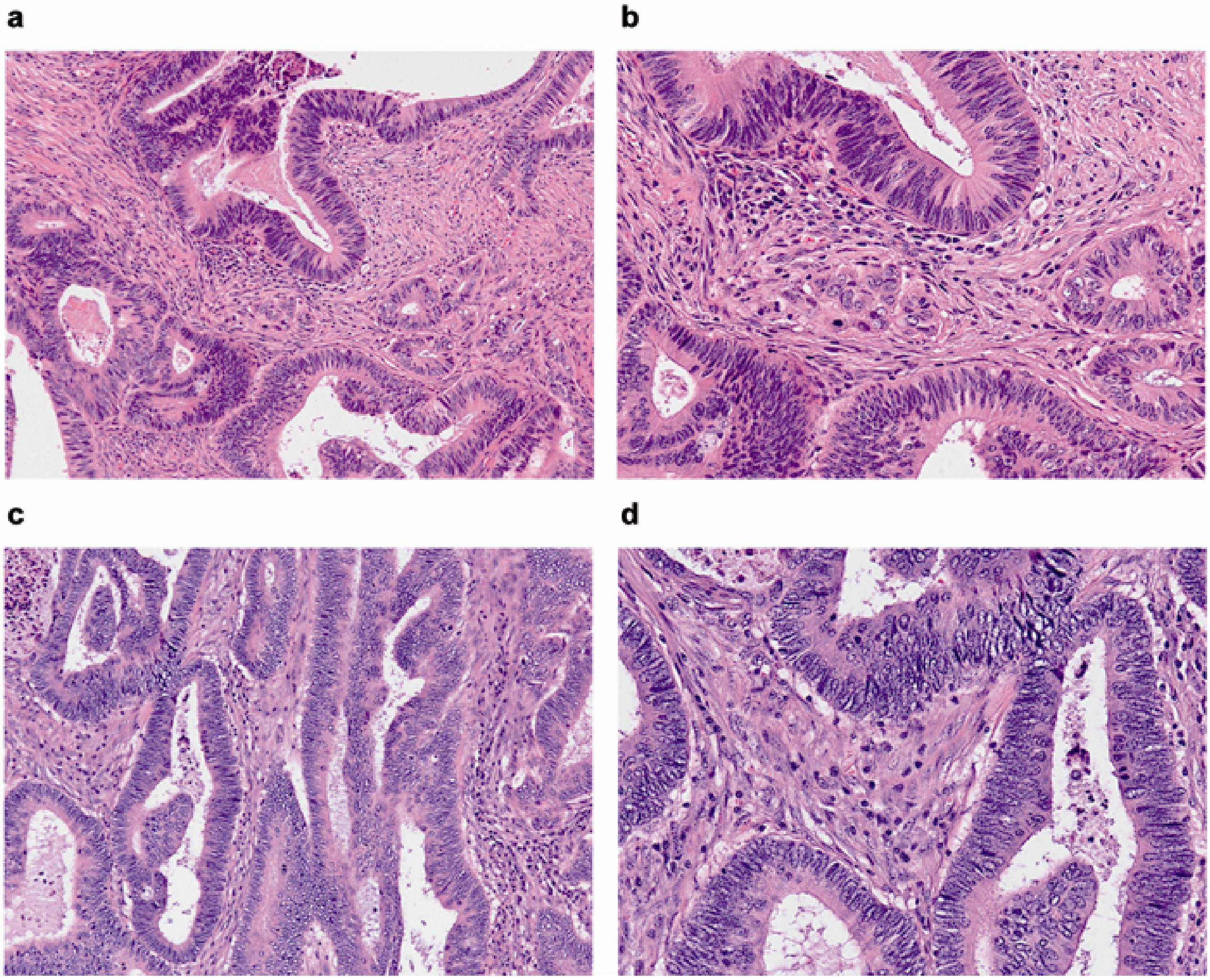

CRC-4

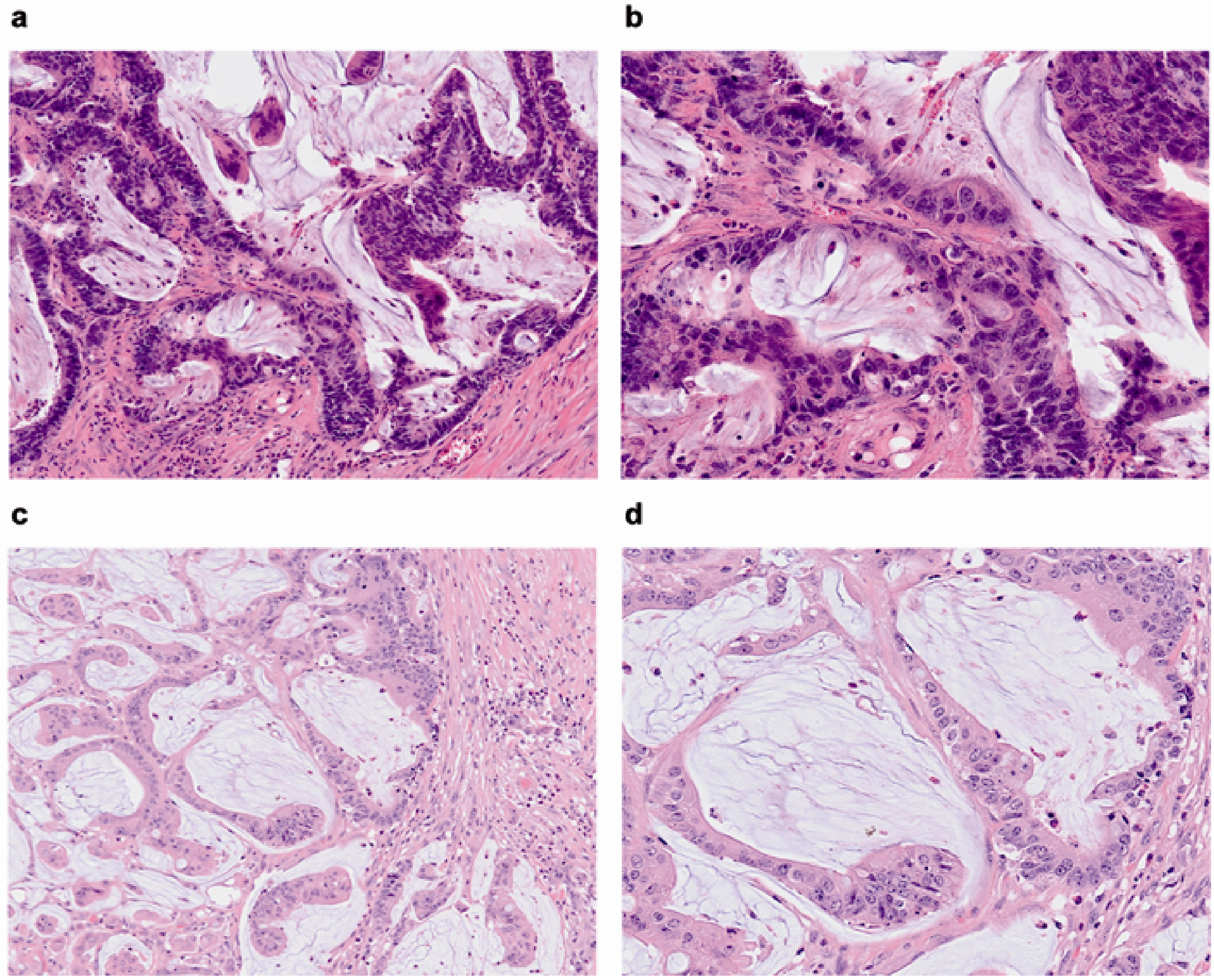

BrCa-1

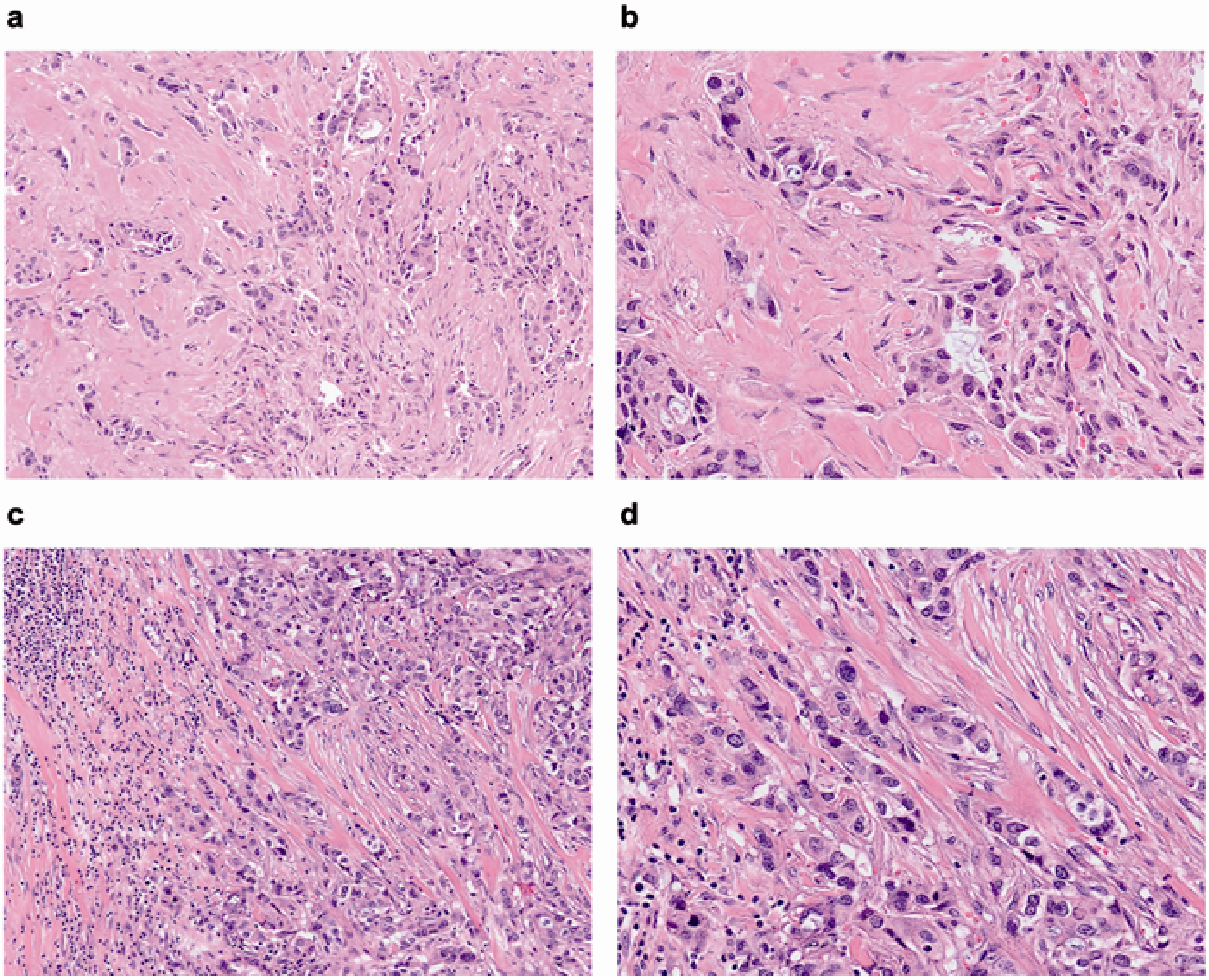

BrCa-2

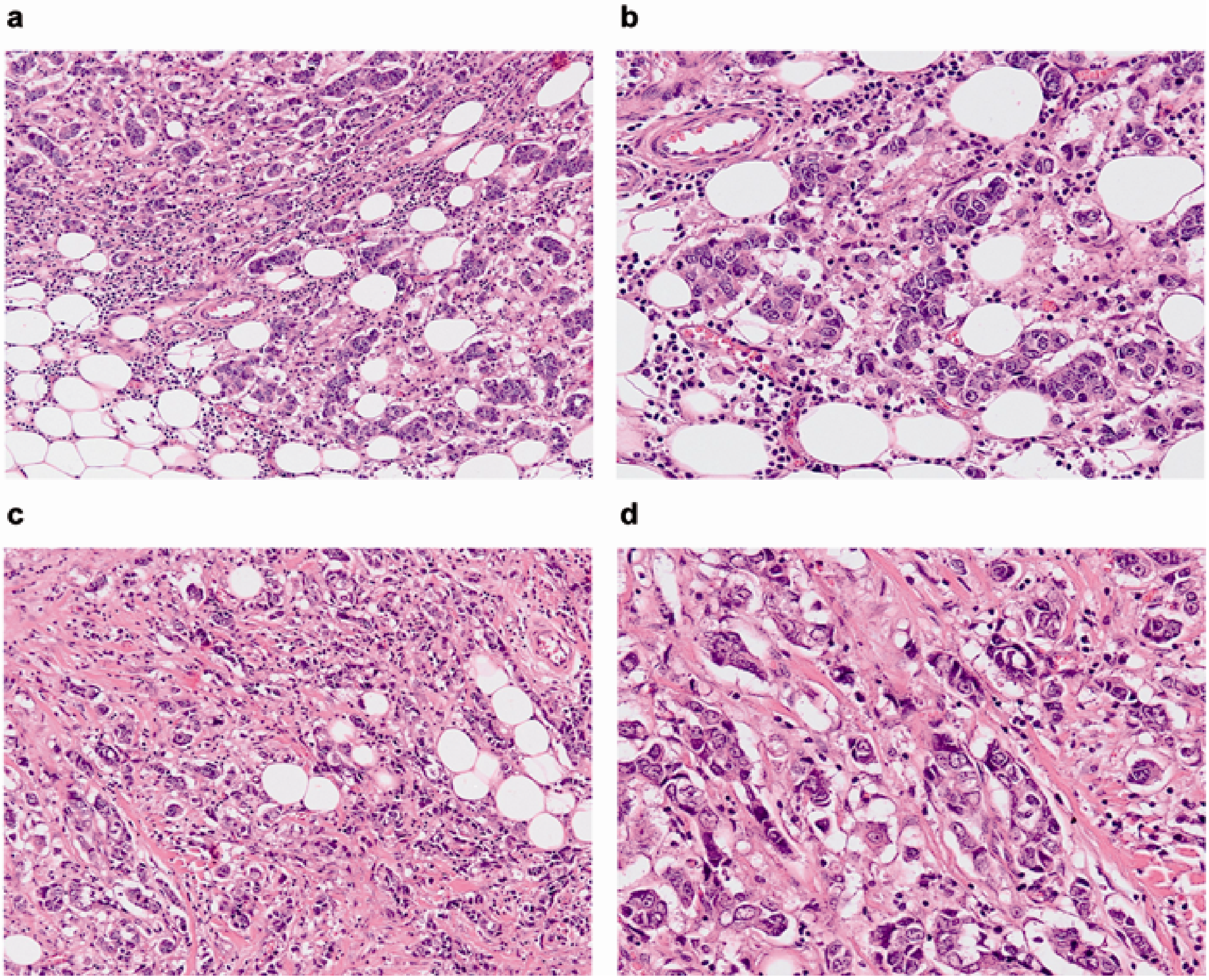

BrCa-3

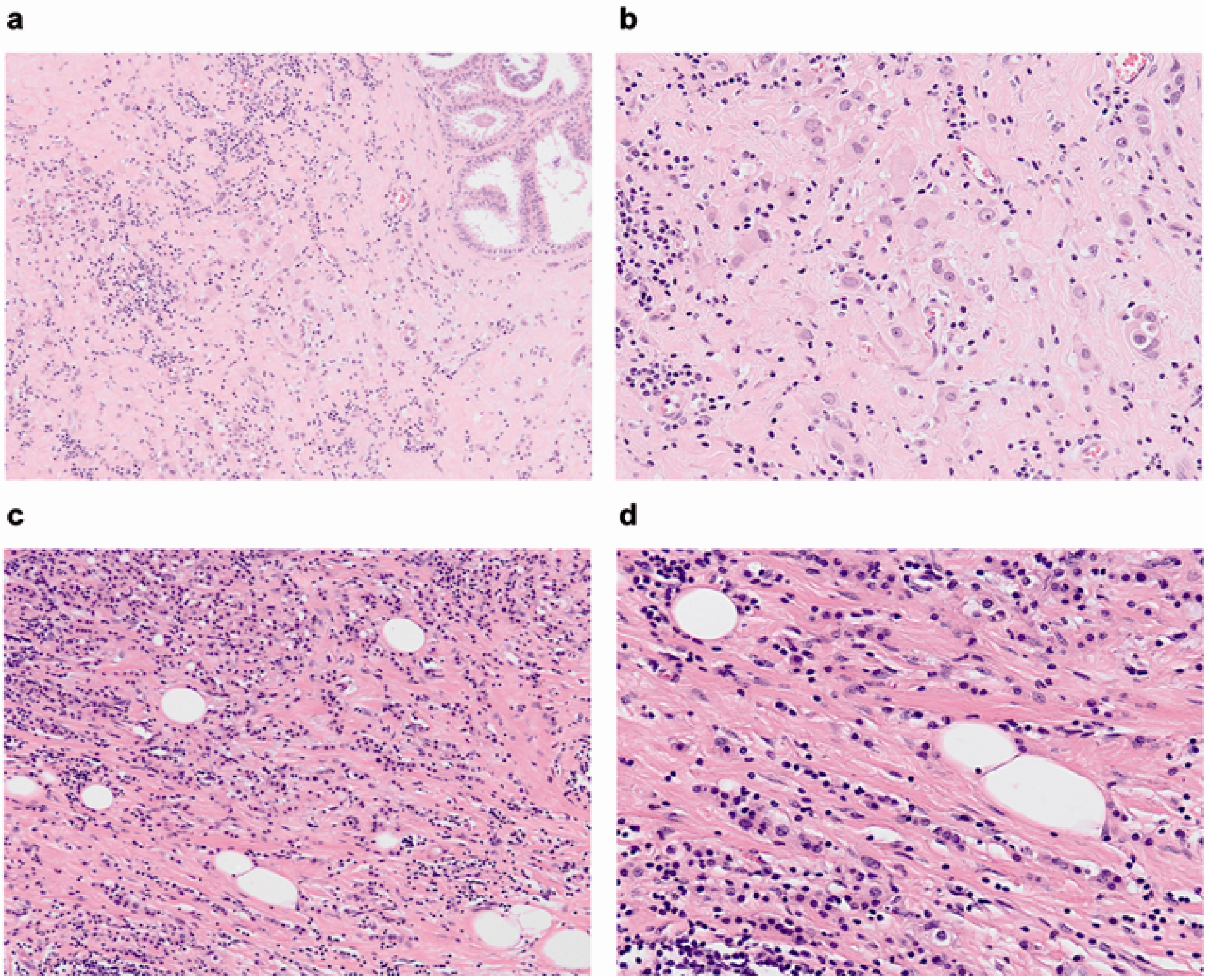

BrCa-4

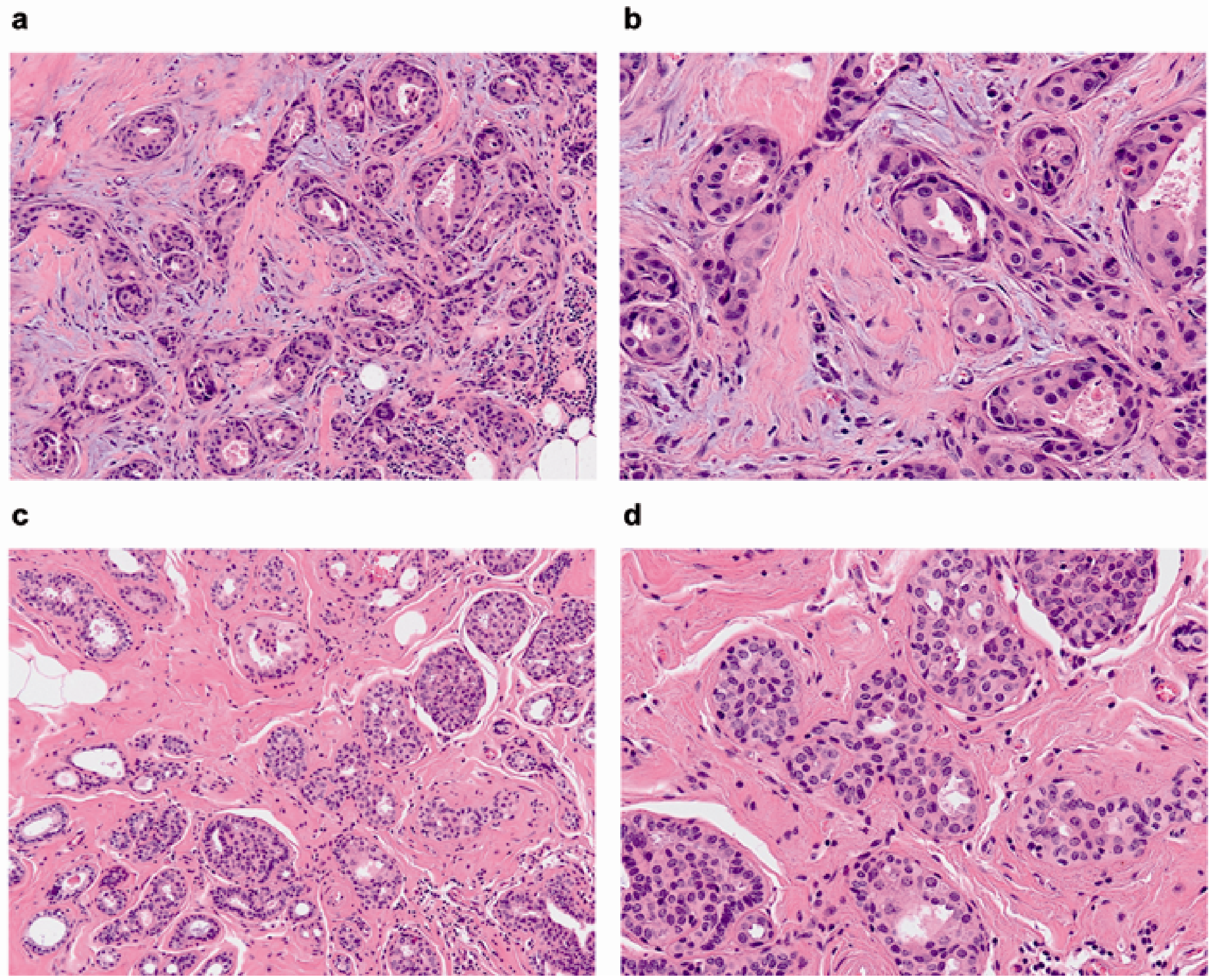

GCa

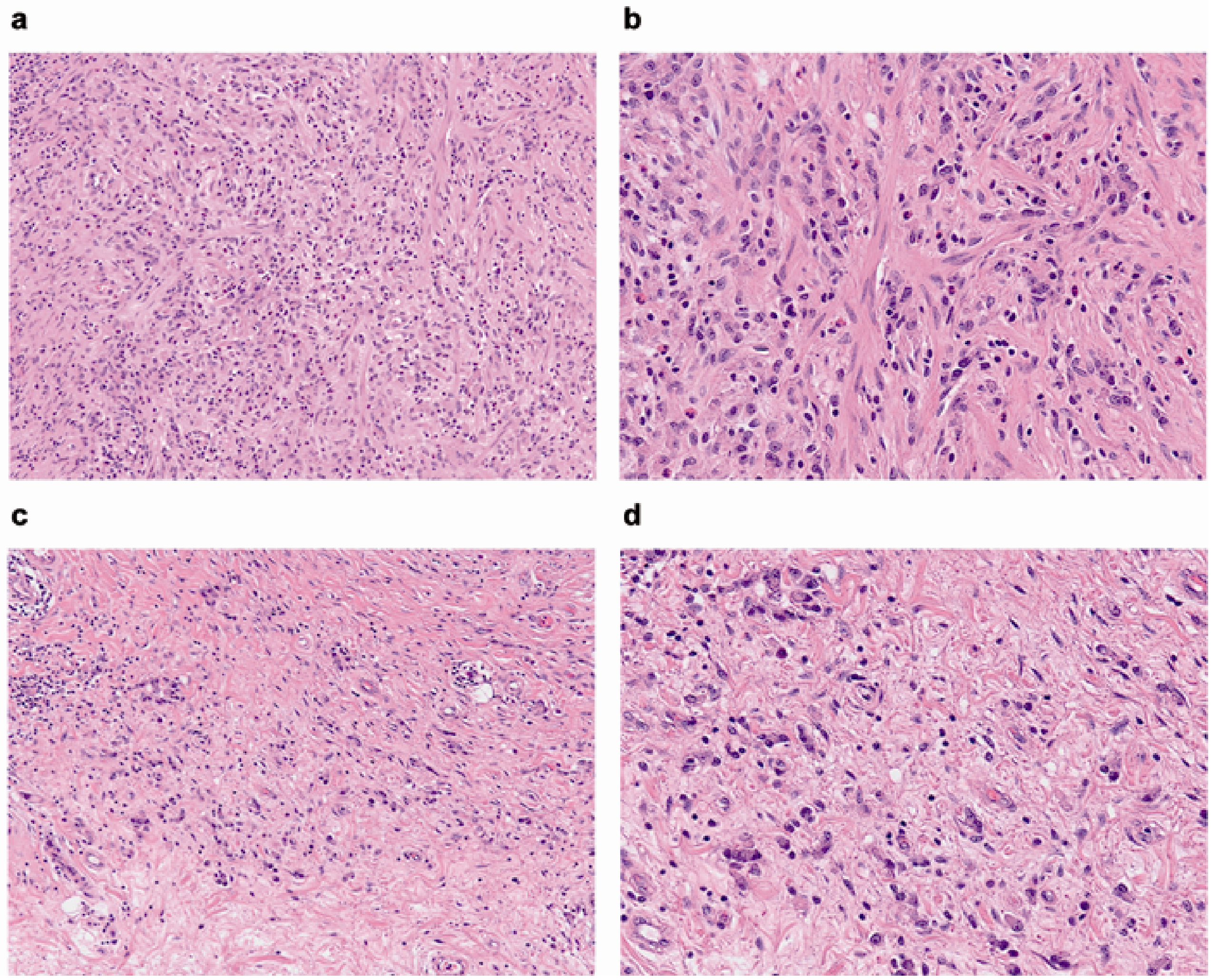

PCa

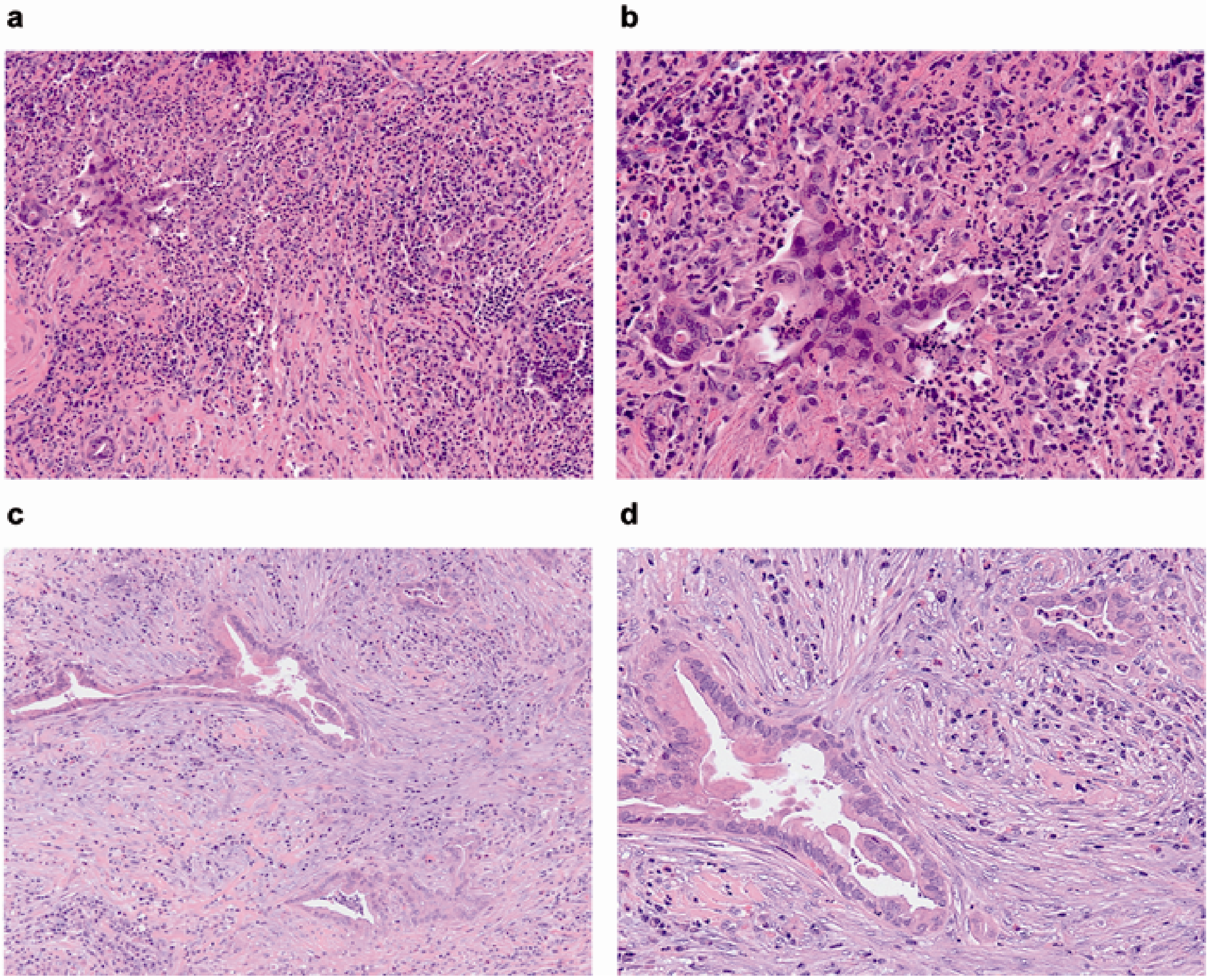

Supplement: Figure S1 — Histology (H&E staining) of tissues processed either routinely (FFPE) or following the CF procedure. For all samples: (a) Cold-Fixed (10×); (b) Cold-Fixed (20×); (c) Standard-Fixed (10×); (d) Standard-Fixed (20×). (PDF) [file pone.0021043.s001.pdf]
