# Supplementary material for: Formalin Fixation at Low Temperature Better Preserves Nucleic Acid Integrity
Source: PLoS One. 2011 Jun 15;6(6):e21043. doi: 10.1371/journal.pone.0021043 (PMC3115967; doi:10.1371/journal.pone.0021043)

Figure S2: Probe detection analysis on cancer samples subdivided by tissue of origin.

(a) Four CRCs; **(b)** Four breast cancers; **(c)** One pancreatic and one stomach cancer. For all CRC samples, a replicate cold fixation was performed and RNA was extracted, to assess consistency of the procedure on the same tissue sample.

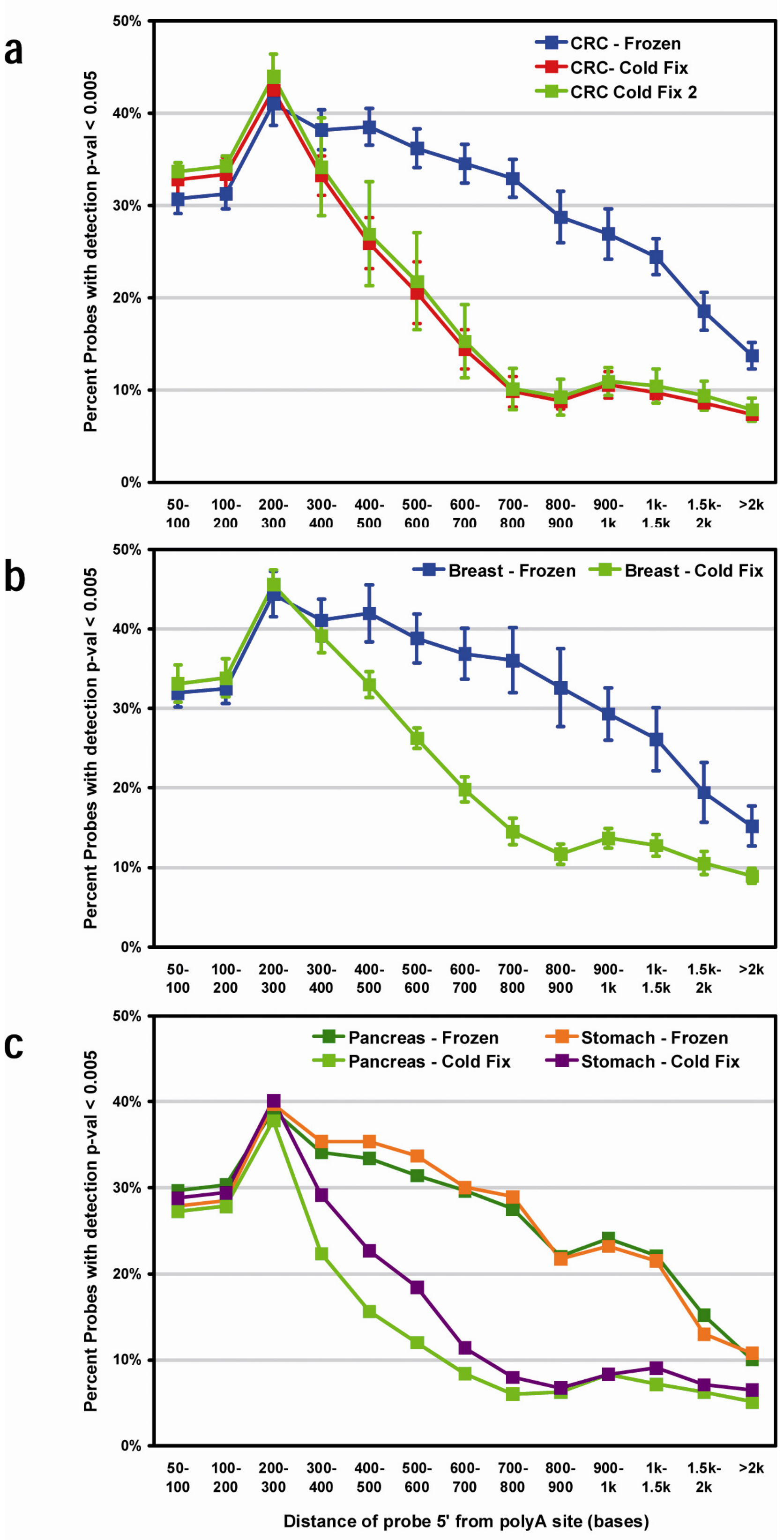

Supplement: Figure S2 — Probe detection analysis on cancer samples subdivided by tissue of origin. (a) Four CRCs; (b) Four breast cancers; (c) One pancreatic and one stomach cancer. For all CRC samples, a replicate cold fixation was performed and RNA was extracted, to assess consistency of the procedure on the same tissue sample. (PDF) [file pone.0021043.s002.pdf]
