# Supplementary material for: Formalin Fixation at Low Temperature Better Preserves Nucleic Acid Integrity
Source: PLoS One. 2011 Jun 15;6(6):e21043. doi: 10.1371/journal.pone.0021043 (PMC3115967; doi:10.1371/journal.pone.0021043)

Figure S3: Cold-fixed samples generate reproducible expression profiles, highly correlated with those generated by frozen samples.

(a) Pearson correlation between frozen (F) and Cold-Fixed (CF) or Standard-Fixed (SF) samples, plus correlation between replicate samples from the same cold-fixed tissue. **(b-e)** Dot plots comparing, expression profiles of RNA from, respectively: frozen vs standard-fixed tissue **(b),** Frozen vs Cold-Fixed tissue of a representative CRC sample **(c),** frozen vs Cold-Fixed tissue of a representative breast cancer sample **(d)**, Cold-fixed vs replicate sample extracted from the same cold-fixed tissue **(e)**.

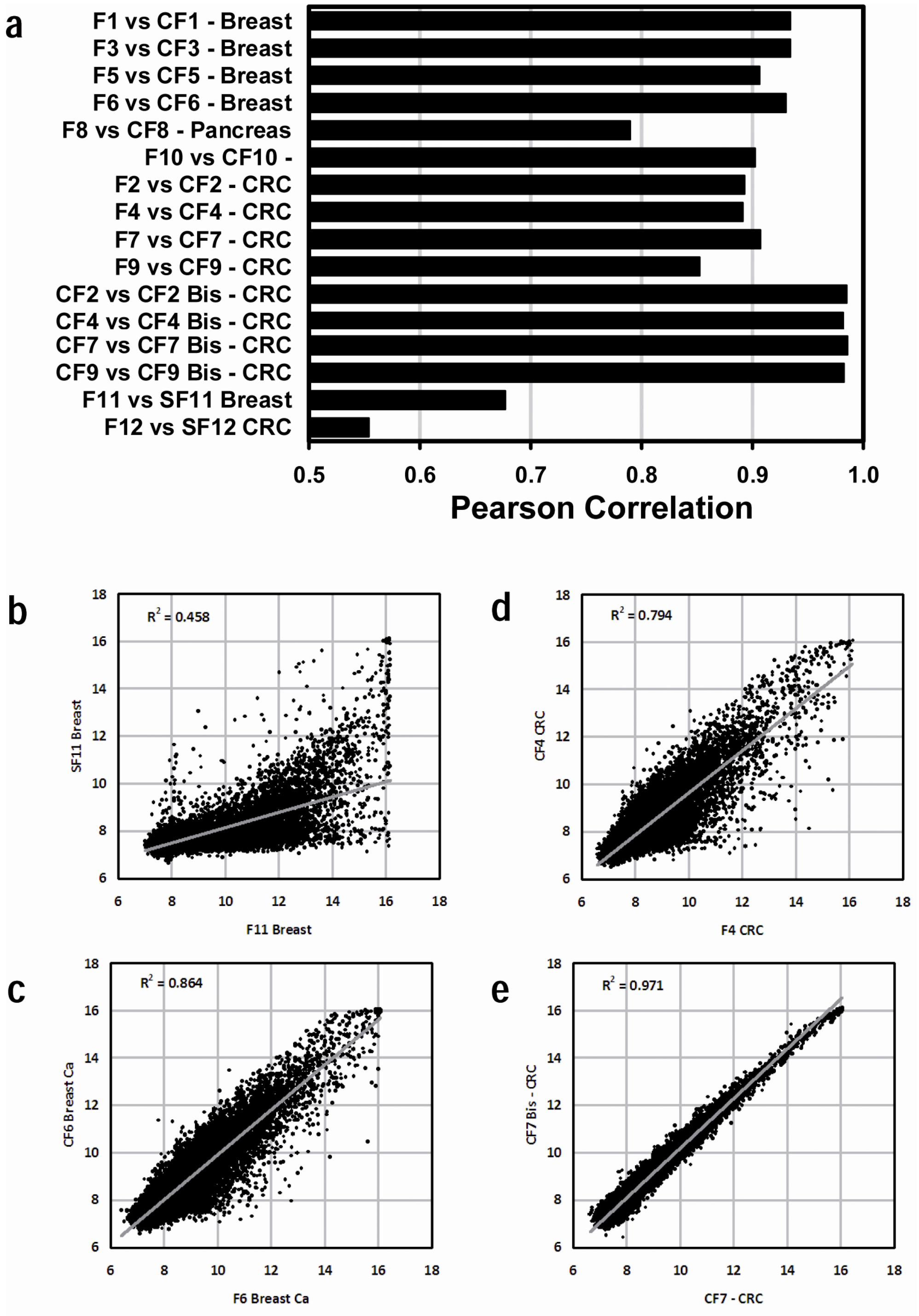

Supplement: Figure S3 — Cold-fixed samples generate reproducible expression profiles, highly correlated with those generated by frozen samples. (a) Pearson correlation between frozen (F) and Cold-Fixed (CF) or Standard-Fixed (SF) samples, plus correlation between replicate samples from the same cold-fixed tissue. (b–e) Dot plots comparing, expression profiles of RNA from, respectively: frozen vs standard-fixed tissue (b), Frozen vs Cold-Fixed tissue of a representative CRC sample (c), frozen vs Cold-Fixed tissue of a representative breast cancer sample (d), Cold-fixed vs replicate sample extracted from the same cold-fixed tissue (e). (PDF) [file pone.0021043.s003.pdf]
