# Supplementary material for: Formalin Fixation at Low Temperature Better Preserves Nucleic Acid Integrity
Source: PLoS One. 2011 Jun 15;6(6):e21043. doi: 10.1371/journal.pone.0021043 (PMC3115967; doi:10.1371/journal.pone.0021043)

Figure S4: RNA from cold-fixed samples generates reliable expression profiles also with probes far away from the Reverse Transcription start site.

Pearson correlation was analyzed for subgroups of microarray probes, based on their distance from the transcript 3'-end. **(a)** Breast Cancer samples; **(b)** CRC samples; **(c)** Pancreas and stomach cancer; **(d)** CRC replicated cold-fixed samples.

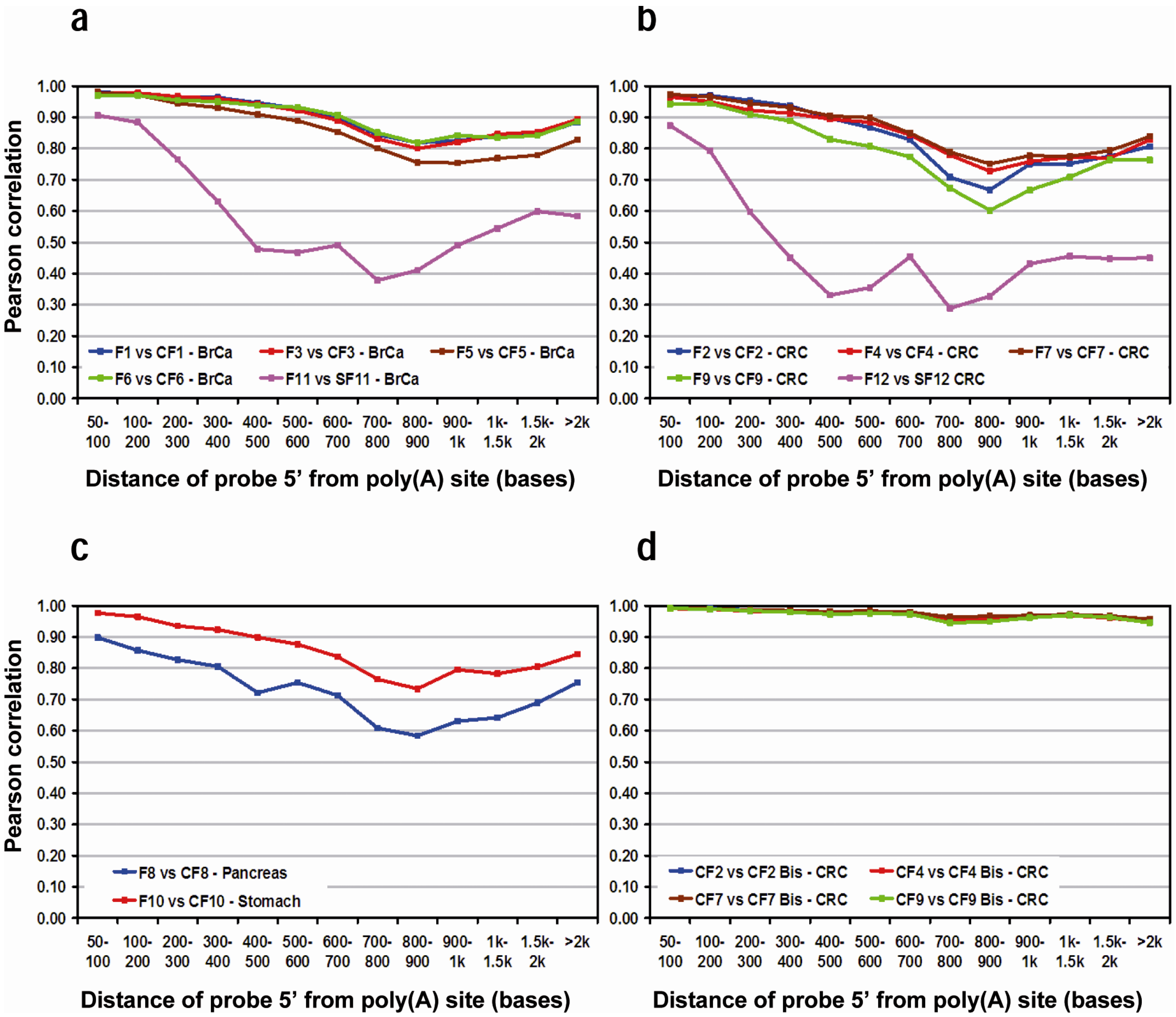

Supplement: Figure S4 — RNA from cold-fixed samples generates reliable expression profiles also with probes far away from the Reverse Transcription start site. Pearson correlation was analyzed for subgroups of microarray probes, based on their distance from the transcript 3′-end. (a) Breast Cancer samples; (b) CRC samples; (c) Pancreas and stomach cancer; (d) CRC replicated cold-fixed samples. (PDF) [file pone.0021043.s004.pdf]
