# Supplementary material for: Formalin Fixation at Low Temperature Better Preserves Nucleic Acid Integrity
Source: PLoS One. 2011 Jun 15;6(6):e21043. doi: 10.1371/journal.pone.0021043 (PMC3115967; doi:10.1371/journal.pone.0021043)
Supplement: Table S1 — RT-PCR conditions. (PDF) [file pone.0021043.s005.pdf]

Table S1: RT-PCR conditions.

Target	Gene	Primer sequences	Size	AT
**cDNA**	*HMBS/ABL1/B2M*	FW *HMBS* 5'- TGAGAGTGATTCGCGTGGGTAC-3'	128 bp	55°C
		REV *HMBS* 5'- CCCTGTGGTGGACATAGCAATG-3'		
		FW *ABL1* 5'- AGCATCTGACTTTGAGCC-3'	193 bp	
		REV *ABL1* 5'- CCCATTGTGATTATAGCCTAAGAC-3'		
		FW *B2M* 5'-CTCGCGCTACTCTCTCTTTCTGG-3'	335 bp	
		REV *B2M* 5'- GCTTACATGTCTCGATCCCACTTAA -3'		
	CK-20	FW1 5'- AGTTCTGCAGCAACAGGTCACAG -3'	329 bp	63.0°C
		FW2 5'- GGAGGAAGTCGATGGCCTACACAA -3'	500 bp	
		FW3 5'- AAATGCTCGGTGTGTCCTGCAAAT -3'	716 bp	
		REV 5'- CTTCCAGAAGGCGGCGGTAAGTAG -3'		
	Mammaglobin SCGB2A2	FW 5'- AGCACTGCTACGCAGGCTCT-3'	331 bp	58.0 °C
		REV 5'-ATAAGAAAGAGAAGGTGTGG-3'		
	ESR1	FW 5'- CTGCTGGCTACATCATCTCG -3'	346 bp	54.0 °C
		REV 5'- TCTCCAGCAGCAGGTCATAG -3'		
**Genomic DNA**	*CK-20*	FW 5'- ATGACCCAAGTTCCAGGGGTGACT -3'		66.0 °C
		REV1 5'- GGTCTGGTCACATGTGGTGTGATG -3'	339 bp	
		REV2 5'- CACAAAGGGCTTTTTGGTGTTAATGC -3'	420 bp	
		REV3 5'- GACAGCAGATGTATGCCACCATGC -3'	755 bp	

Size = Size of PCR product; **AT** = Annealing temperature.
